# ZIPK activates the IL‐6/STAT3 signaling pathway and promotes cisplatin resistance in gastric cancer cells

**DOI:** 10.1002/2211-5463.13270

**Published:** 2021-08-22

**Authors:** Haonan Fan, Qifeng Ou, Qiao Su, Guanman Li, Zhijuan Deng, Xiaohui Huang, Jiong Bi

**Affiliations:** ^1^ Laboratory of General Surgery The First Affiliated Hospital Sun Yat‐sen University Guangzhou China; ^2^ Laboratory Animal Center The First Affiliated Hospital Sun Yat‐sen University Guangzhou China; ^3^ School of Medicine (Shenzhen) Sun Yat‐sen University Guangzhou China; ^4^ Ultrasound Medical Center The First people’s Hospital of Chenzhou Chenzhou China

**Keywords:** chemotherapy resistance, gastric cancer, zipper interacting protein kinase, IL‐6/STAT3 signaling pathway

## Abstract

Gastric cancer is one of the most common malignant cancers globally. Chemotherapy resistance remains a major obstacle in the treatment of gastric cancer, and the molecular mechanisms underlying drug resistance are still not well understood. We previously reported that Zipper interacting protein kinase (ZIPK), also known as death‐associated protein kinase3, exerts an oncogenic effect on gastric cancer via activation of Akt/NF‐κB signaling and promotion of stemness. Here, we explored the roles of ZIPK in cisplatin resistance. We report that ZIPK enhances cell proliferation and invasion and reduces the antitumor activity of cisplatin in gastric cancer. In addition, our western blot data suggest that ZIPK activated the IL‐6/STAT3 signaling pathway. Furthermore, ZIPK increased the expression of IL‐6 and multidrug‐resistance genes. Using the STAT3 inhibitor stattic to block the IL‐6/STAT3 signaling pathway strongly increased the sensitivity of ZIPK‐expressed cells to cisplatin. In conclusion, ZIPK may play a role in cisplatin resistance through activation of the IL‐6/ STAT3 signaling pathway. Inhibition of STAT3 in gastric cancer overexpressing ZIPK might have potential to improve the efficacy of cisplatin.

## Graphical abstract

ZIPK activates the IL‐6/STAT3 signaling pathway via Ser727 phosphorylation in gastric cancer cells and increases IL‐6‐induced STAT3‐dependent transcription. Effective blockade of STAT3 activity by the STAT3 inhibitor stattic sensitized gastric cancer cells to cisplatin and reduced expression of the cisplatin resistance‐related genes *G3BP2*, *THOC1*, *ATP7A*, and *OTUD1*.

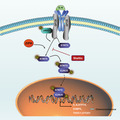

**Abbreviations**
DAPK: death‐associated protein kinase
DCS therapy: S‐1, cisplatin, and docetaxel combination chemotherapy
IL‐6: interleukin 6
STAD: stomach adenocarcinoma
TCGA: the cancer genome atlas
ZIPK: zipper interacting protein kinase

Gastric cancer (GC) is one of the most common malignant cancers worldwide. The highest estimated incidence and mortality rates are in eastern Asia, particularly in Korea, Mongolia, Japan, and China. Surgery is the curative therapy for early‐stage gastric cancer. Systemic chemotherapy with multiple drug regimens is used in the treatment of advanced GC. However, the development of drug resistance restricts the effectiveness of chemotherapy and results in treatment failure [1, 2, 3]. Cisplatin (CDDP) is one of the first‐line chemotherapeutic agents for GC. Cisplatin exerts its antineoplastic effect through inducing unrepairable DNA lesions, which can lead to a proliferative arrest or mitochondrial apoptosis [4, 5, 6, 7]. Cancer cells continuously exposed to CDDP often develop multiple mechanisms to overcome CDDP‐induced apoptosis [8, 9]. Individual variations among patients and genetic heterogeneity among tumor cells contribute to CDDP resistance [10, 11]. Although numerous studies have reported that decreased drug uptake, increased drug efflux, increased DNA damage repair, alterations in apoptotic signaling pathways occur in the presence of CDDP resistance, the precise mechanisms are not well understood so far [12, 13]. Therefore, clarifying the molecular mechanisms of CDDP resistance is crucial for improving gastric cancer survival.

Zipper interacting protein kinase (ZIPK), also known as death‐associated protein kinase3, is a serine/threonine kinase that mediates a variety of cell functions, including apoptosis, autophagy, adherence, and cell proliferation [14]. ZIPK has been regarded as a tumor suppressor because of its inhibitory role in carcinoma development through the pro‐apoptosis and pro‐autophagy function [15, 16, 17]. On the other hand, some recent studies demonstrate that ZIPK can promote cell growth, proliferation, and invasion in lung cancer and prostate cancer [18, 19]. Our previous study showed that ZIPK exerted its oncogenic effect on gastric cancer cells via activation of Akt/NF‐κB signals and promotion of stemness [20]. It is well known that cancer stem cells are resistant inherently to chemotherapy and enriched in residual lesions after therapy, so cancer stem cells are considered as the culprits of tumor recurrence and metastasis [21]. Based on our previous finding that ZIPK may enhance the stemness of cancer cells, we investigated the effect of ZIPK on chemotherapy in the present study. We found that ZIPK expression decreased the sensitivity of gastric cancer cell to cisplatin. The further finding suggests that ZIPK increased the activity of IL‐6/ STAT3 signaling pathway, leading to CDDP resistance. Using the STAT3 inhibitor dramatically sensitized the ZIPK‐overexpressed cells to cisplatin. Our study may provide a promising new therapeutic target for gastric cancer treatment.

## Method

### Cell lines

HGC27 and SGC7901 were kindly provided by Professor Jie Chen from the Department of Gastroenterology, the First Affiliated Hospital of Sun Yat‐sen University. These cell lines were cultured in DMEM with 10% fetal bovine serum and 1% penicillin/streptomycin.

### Lentivirus and reagents

Lentiviral vectors encoding ZIPK, short hairpin RNAs (shRNAs) against ZIPK, and the empty vector (LV‐vector and ‐shNC) were purchased from GeneChem (GeneCopoeia, MD). To produce infectious lentiviral particles, human embryonic kidney 293FT (HEK293FT) cells were cotransfected with each expression vector and the packaging plasmids in the Lenti‐Pac HIV packaging mix (GeneCopoeia, MD). Viral supernatants were harvested after transfection for 2 days and then added to cell culture. After infection, cells were selected in 1 μg·mL^−1^ puromycin for 14 days.

### Cell proliferation

Cell proliferation was detected by using the cell counting Kit‐8 (CCK‐8) assay (Dojindo; Japan). 1 × 10^3^ cells were plated in 96‐well. Cell proliferation was measured once a day until day 5. According to the manufacturer's guidelines, 10 μL CCK8 reagent was added in each well and incubated in the dark for 2 h at 37 . Absorbance at 450 nm was analyzed using a microplate reader.

### Wound‐healing assay

Cells in the logarithmic growth phase were plated in 6‐well plates at the appropriate density. Until confluent, wounds were made using a 200 μL pipette tip. After scratching, migration photographs were captured at 0 and 48 h.

### Transwell assay

For transwell assay, cells (4 × 10^4^) in DMEM medium with 2% FBS were placed in the upper chamber, and a medium containing 15% FBS was added to the lower chamber. The chambers were then incubated for different time points for cell migration (without matrigel) and invasion (with 250 μg·mL^−1^ matrigel coating) at 37 °C. After incubation, the cells were fixed with 50% methanol and stained with 0.1% crystal violet. Pictures of cells were taken in 10 fields under a 10 × objective lens.

### Cell viability

Briefly, cells were seeded in a 96‐well plate at a density of 5000 cells/well, cultured overnight, and treated with different cisplatin concentrations for 24 h. After that, the OD value was measured, and the cell death rate at each dose was calculated.

### Colony formation assay

Cells seeded in separate wells of a 6‐well plate at a density of 1000 cells/well were cultured overnight and treated with cisplatin or cisplatin combined with stattic for 2 weeks. After that, crystal violet was used to stain the surviving colonies (> 50 cells per colony).

### Trypan blue staining assay

Cell viability also was assessed by trypan blue staining. Cells treated with different doses of cisplatin were diluted to the desire concentration. 90 μL cell suspension mixed with 10 μL trypan blue, and the cells were counted. The results represent the percentage of cell death (%) with respect to the control group.

### Qualitative real‐time PCR analysis

Total RNA from cells was extracted using TRIzol reagent. Extracted RNA was reverse transcribed to cDNA by Takara PrimeScript TMRT reagent kit. The real‐time quantitative PCR reaction was performed with the SYBR green detection system. GAPDH served as an endogenous reference. RT‐PCR was performed on LightCycle480 II. The primer pairs for each target gene are listed in Table S1. All experiments were performed in triplicates.

### Western blot

An appropriate extracted buffer was added in cultured cells, and soluble protein was separated from cell lysates by centrifuging the cells at 12 000 × ***g*** for 15 min 4 °C. The protein concentration was determined using the BCA Protein Assay Kit. The proteins in each group were resolved on 10% SDS/PAGE and then transferred to a PVDF membrane (Millipore). After blocking with 5% nonfat milk for 2 h at room temperature, the membranes were incubated with different primary antibodies overnight at 4 . After washing with TBS‐T, membranes were incubated for 2 h with appropriate secondary antibodies. Immunoreactive bands were detected by the ECL (EMD Millipore, MA, USA) method. GAPDH was used as a loading. The information about antibodies was listed in Table S2.

### Weighted correlation network analysis for discovering drug resistance‐related gene modules

RNA‐seq data of 407 STAD (STAD‐TCGA) patients were downloaded from TCGA (https://portal.gdc.cancer.gov/), and a transcription gene expression matrix was extracted. The correlation between the mRNA expression level of STAT3 and 34 532 different transcripts was calculated by Spearman. Genes with the absolute value of the correlation coefficient greater than 0.3 and the *P*‐value of less than 0.05 were considered to have a high relationship to STAT3 expression. Differentially expressed genes were identified between patients of advanced gastric cancer with sensitivity or resistance to S‐1, cisplatin, and docetaxel combination chemotherapy (DCS therapy), based on public GEO data (GSE31811) using the limma package in R [22]. STRING (https://string‐db.org/) was employed to construct a protein–protein interaction (PPI) network.

### Statistical analysis

Statistical analysis was conducted using graphpad prism 8. The data from cell growth, wound healing, transwell, cell viability, western blot, and qPCR assays were evaluated through Student's *t*‐test. The value of *P* < 0.05 was considered statistically significant.

## Results

### ZIPK promoted the tumorigenic ability of gastric cancer cells and reduced antitumor activity of cisplatin

**FIGURE 1:**
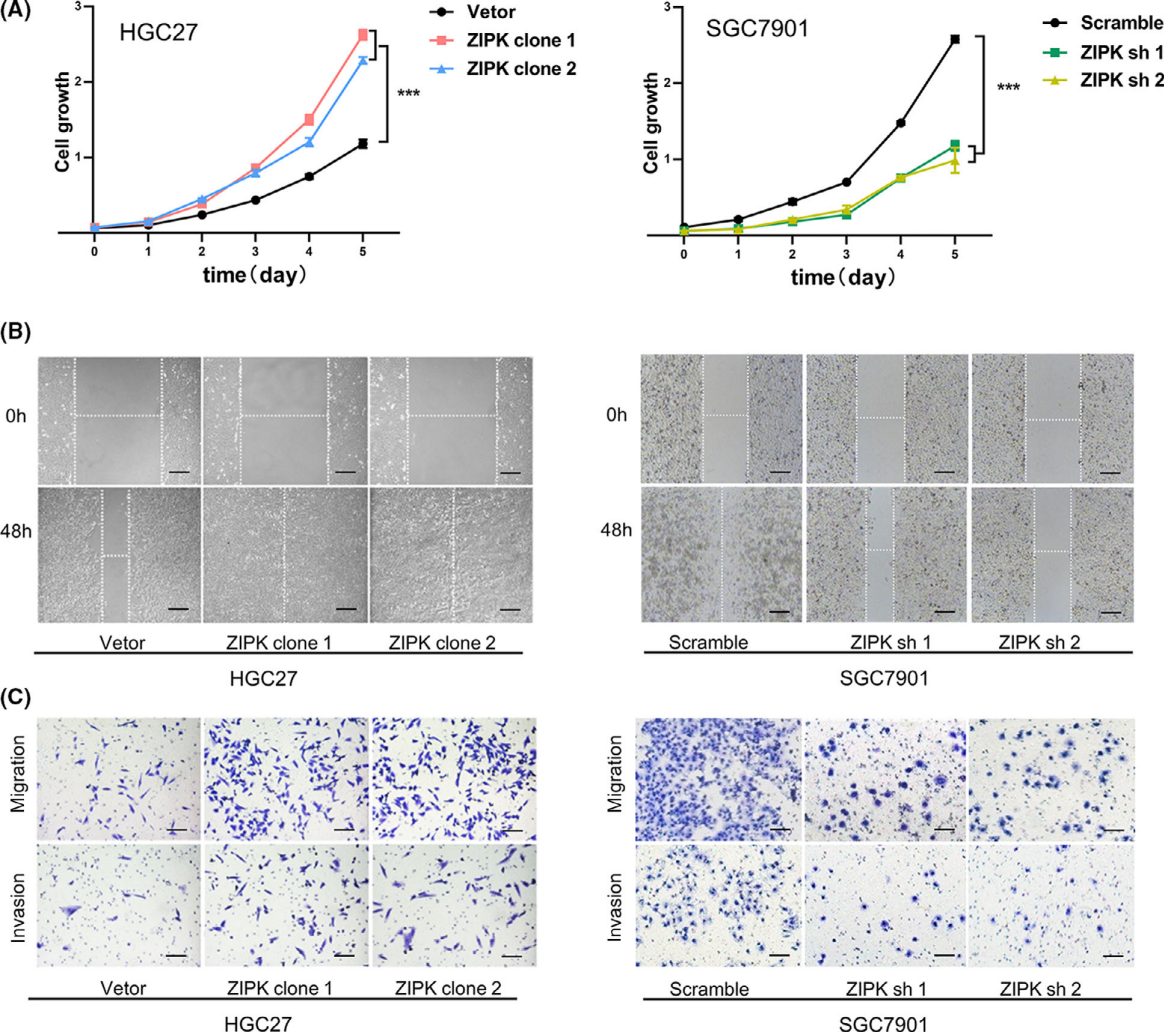
ZIPK promoted the tumorigenic ability of gastric cancer cells. Cell proliferation, invasion, and migration in HGC27 and SGC7901 cells were analyzed with CCK‐8 assay (A), Transwell assay (B), and wound‐healing assay (C). The results were expressed as the mean ± SD (***indicates *P* < 0.001 in Student’s *t*‐test) (Scale bars: 100 μm)

**FIGURE 2:**
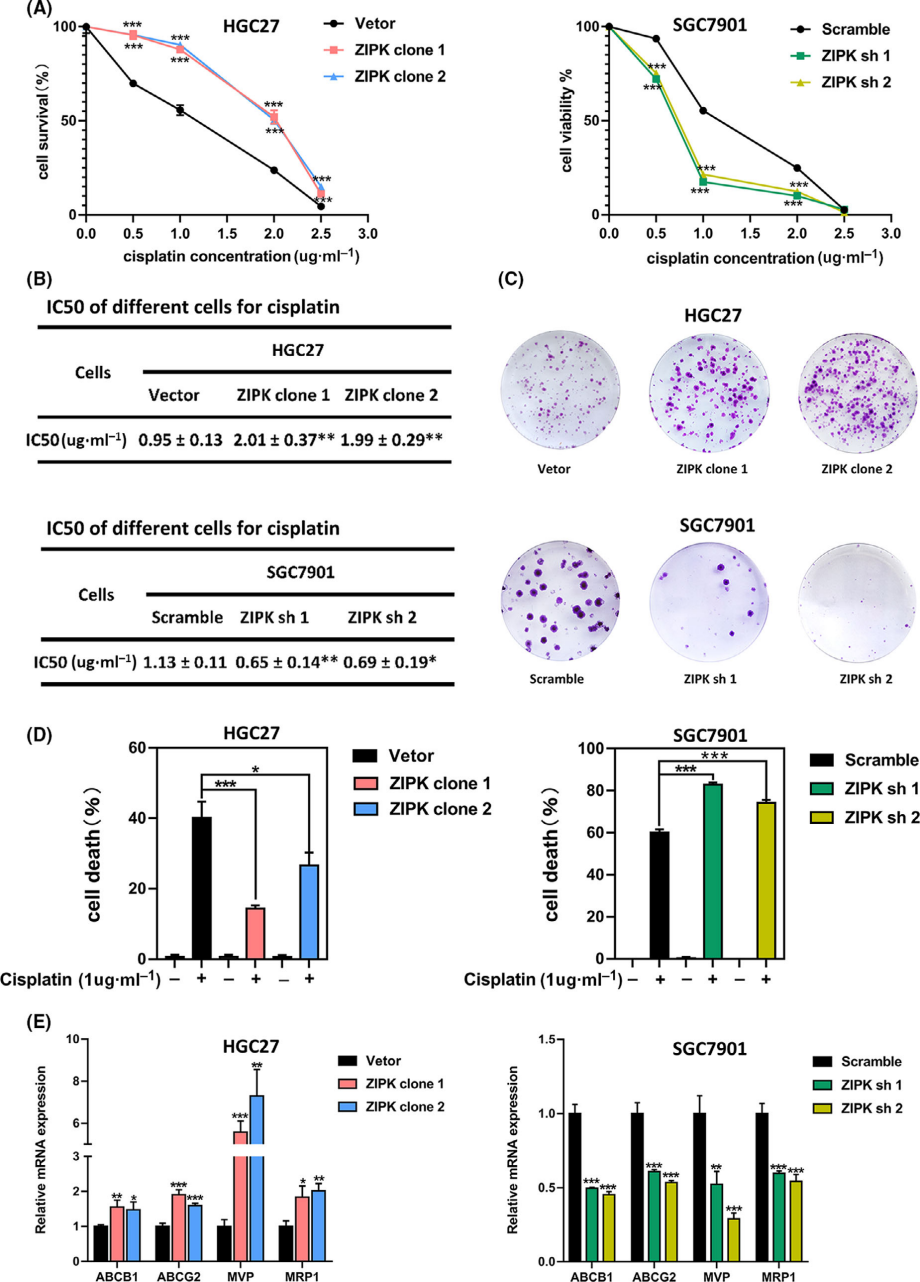
ZIPK reduced the antitumor activity of cisplatin in gastric cancer cells. (A) GC cells were treated with cisplatin at different concentrations for 24 h and cell survival was measured by CCK‐8 assay. Three independent assays were conducted. The results were expressed as the mean ± SD (*, *P* < 0.05; **, *P* < 0.01; ***, *P* < 0.001, Student’s *t*‐test). (B) The evaluation of IC50 to cisplatin in HGC27 and SGC7901 cells after 24 h. (C) Cell viability was determined by colony formation assay after cisplatin treatment for 2 weeks. (D) Cell death was determined by trypan blue staining assay after 24‐h treatment with cisplatin (1 μg·mL^−1^). (E) qPCR analysis of gene expression in ZIPK‐overexpressed, ZIPK‐silenced, and their respective control cells. Three independent assays were performed. The results were expressed as the mean ± SD (*, *P* < 0.05; **, *P* < 0.01; ***, *P* < 0.001, Student’s *t*‐test)

Our previous study revealed that ZIPK increased cancer cell EMT, metastasis, and stemness [20]. It is accepted that cancer cells acquiring stemness features and mesenchymal phenotype are resistant to chemotherapy drugs, so we proposed that ZIPK may have an effect on chemosensitivity in GC cells. According to our previous experimental results [15], we chose high ZIPK expressed SGC7901 cells and low ZIPK expressed HGC27 cells for studying the roles of ZIPK in gastric cancer. ZIPK expression was stably upregulated in HGC27 cells and silenced in SGC7901 cells by lentiviral transduction, as previously reported (Fig. S1) [17, 20]. Then, functional assays were performed to ascertain the pro‐oncogenic roles of ZIPK in HGC27 and SGC7901cells. Cell counting Kit‐8 (CCK8) assay showed that overexpression of ZIPK markedly enhanced cell proliferation, whereas knockdown of ZIPK inhibited cell proliferation (Fig. 1A). Wound‐healing and transwell assays indicated that the migratory and invasive abilities were significantly increased in ZIPK‐overexpressed cells and decreased in ZIPK‐silenced cells, compared with their control cells respectively (Fig. 1B,C). To identify the effect of ZIPK expression in regulation of cisplatin (CDDP) sensitivity, we tested growth inhibition of HGC27 and SGC7901 cells treated with CDDP at different concentration by CCK‐8 assay. The half maximal inhibitory concentration (IC50) of HGC27 cells to CDDP was 0.89 mg·L^−1^, and IC50 of SGC7901 cells was 0.94 mg·L^−1^ (Fig. S2, *P* < 0.05). The gastric cancer cells with ZIPK overexpression were resistant to CDDP. Conversely, knockdown of ZIPK increased sensitivity to CDDP in gastric cancer cells (Fig. 2A). Compared with the control cells, the IC50 to CDDP was elevated in ZIPK‐overexpressed cells and reduced in ZIPK‐silenced cells (Fig. 2B). Meanwhile, we also detected cell viability by colony formation and measuring membrane permeabilization to trypan blue. Colony formation assay showed that a higher number of foci and larger colonies were yielded (*P* < 0.01) in the ZIPK high‐expressed cells treated with or without CDDP (Fig. 2C). Similarly, trypan blue staining assay showed that ZIPK significantly attenuated CDDP‐induced cell death (Fig. 2D). Next, we examined the mRNA levels of multidrug‐resistance genes in gastric cancer cells. As expected, ZIPK induced multidrug‐resistance genes expression (Fig. 2E). These results supported our hypothesis that ZIPK contributed to chemotherapy resistance development.

### ZIPK activated IL‐6/STAT3 signaling pathway in gastric cancer cells

**FIGURE 3:**
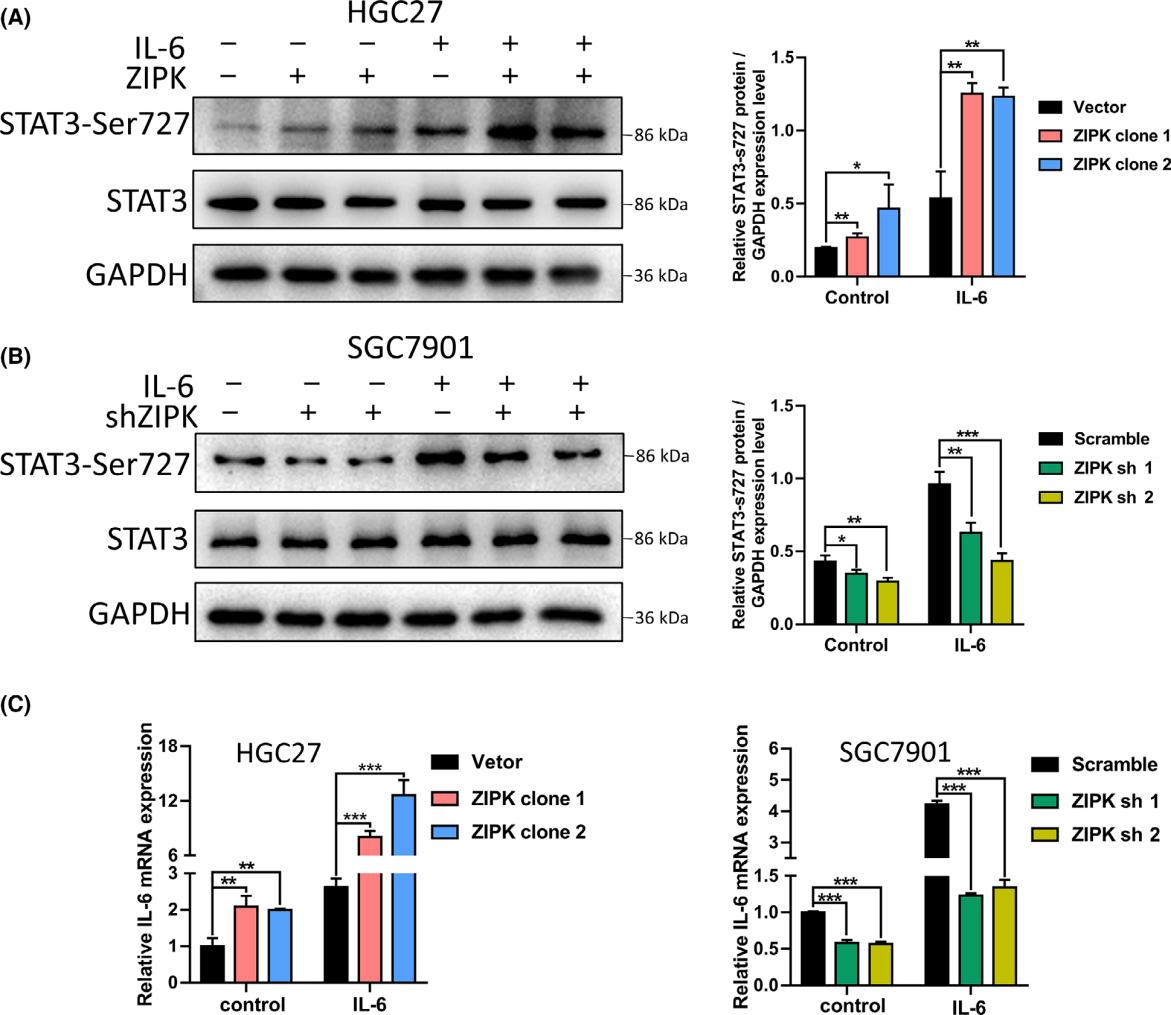
ZIPK activated IL‐6/STAT3 signaling pathway in gastric cancer cells. ZIPK‐overexpressed, ZIPK‐knockdowned, and their respective control cells were incubated with or without IL‐6 (50 ng·mL^−1^) for 30 min. The protein levels of STAT3‐Ser727 and STAT3 were detected by western blot. GAPDH was used as a loading control (A, B). (C) Cells were treated with IL‐6 for 30 min. The expression of IL‐6 was tested by quantitative real‐time PCR. The results were expressed as the mean ± SD (*, *P* < 0.05; **, *P* < 0.01; ***, *P* < 0.001, Student’s *t*‐test; data are means ± SD)

Aberrant hyperactivation of IL‐6/STAT3 signaling occurs in many types of cancer and drives the proliferation, survival, invasiveness, and metastasis of tumor cells [23]. A series of study reveals that IL‐6/STAT3 pathway has a key role in drug resistance [24]. ZIPK has been identified as a STAT3 upstream kinase to phosphorylate STAT3 at Ser727 and enhance IL‐6‐induced STAT3 transcription [25]. In this study, we investigated whether ZIPK promoted drug resistance through activation of IL‐6/STAT3 pathway. Western blot assay showed phosphorylation of STAT3 at Ser727 was significantly increased in ZIPK‐overexpressed cells, and the levels of Ser727 phosphorylation were further elevated after IL‐6 treatment (Fig. 3A). Conversely, knockdown of ZIPK dramatically weakened the phosphorylation levels of STAT3 (Fig. 3B). We noticed that the increase of the phosphorylation levels of STAT3 was more remarkable than that of STAT3 protein levels, indicating that ZIPK regulated STAT3 via post‐translational modification (Fig. 3,A,B). Based on the finding that STAT3 binds to the IL‐6 promoter and leads to increased IL‐6 expression [26], we examined the mRNA levels of IL‐6 by qPCR in ZIPK‐overexpressed, ZIPK‐silenced, and their control cells. Similarly, ZIPK accelerated the mRNA levels of IL‐6 (Fig. 3C). These observations indicated that ZIPK could enhance IL‐6/STAT3 signaling pathway.

### Inhibition of STAT3 increased the sensitivity of ZIPK‐expressed gastric cancer cells to cisplatin

**FIGURE 4:**
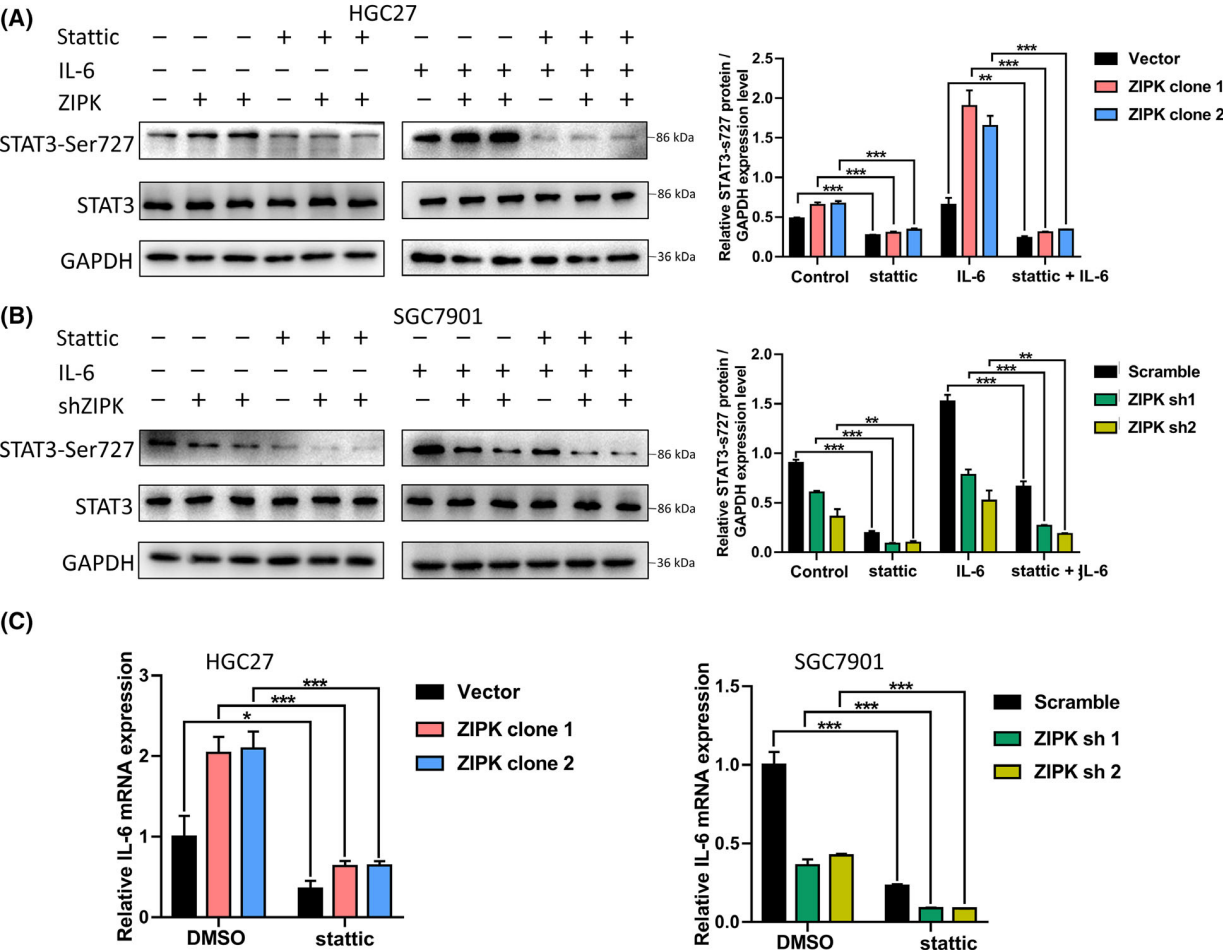
Stattic inhibited IL‐6/STAT3 signaling pathway in gastric cancer cells. The protein levels of STAT3‐Ser727 phosphorylation and STAT3 in ZIPK‐overexpressed, ZIPK‐knockdowned, and their respective control cells with or without exposure to IL‐6 were detected by western blot after static treatment. GAPDH was used as a loading control (A, B). (C) The expression of IL‐6 was tested by quantitative real‐time PCR. The cells were treated with stattic for 24 h. The expression of IL‐6 was tested by quantitative real‐time PCR. The results were expressed as the mean ± SD (*, *P* < 0.05; **, *P* < 0.01; ***, *P* < 0.001, Student’s *t*‐test)

**FIGURE 5:**
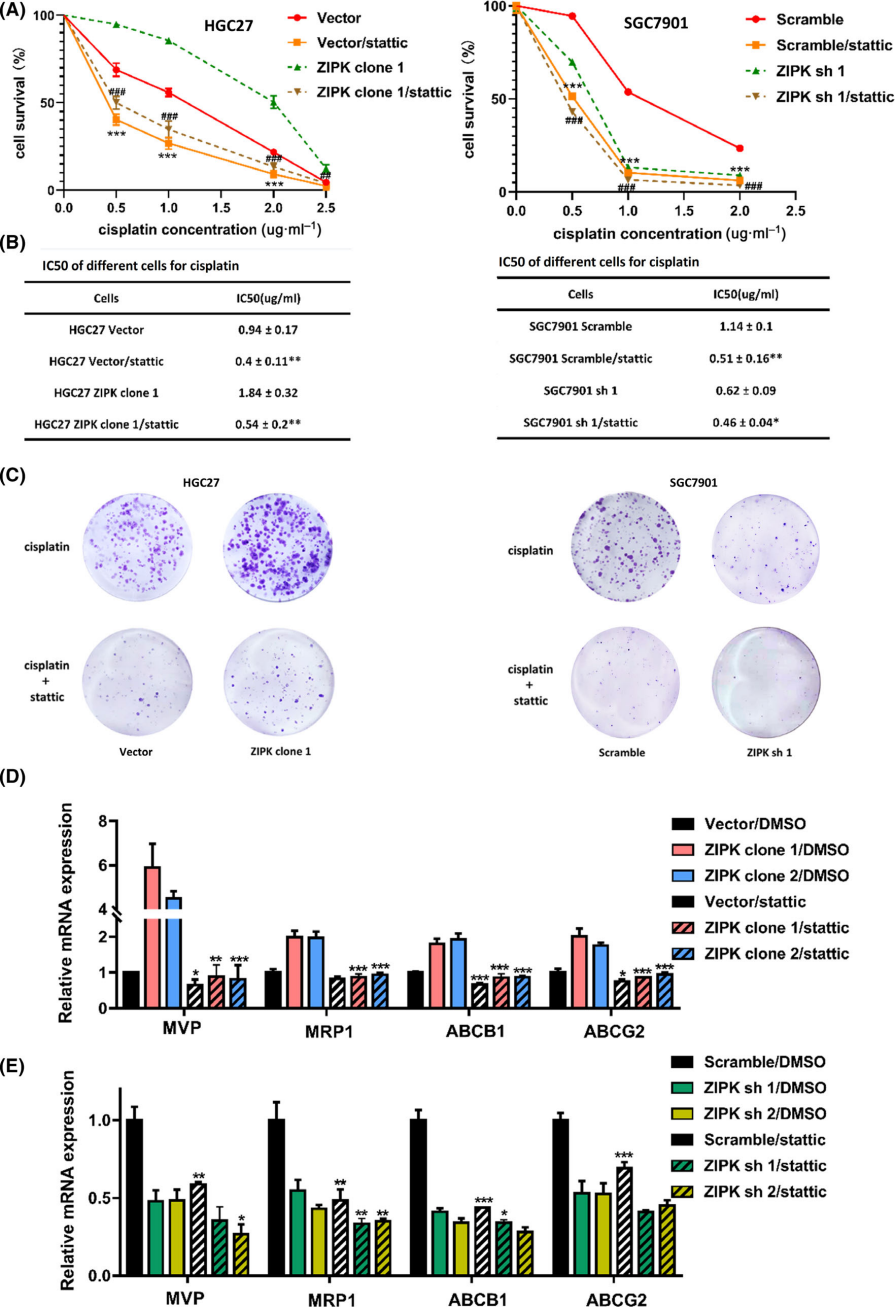
Stattic increased the sensitivity of ZIPK‐expressed gastric cancer cells to cisplatin. (A) The indicated cells were treated with cisplatin, stattic or combination of cisplatin and static. Cell viability was measured by the CCK8 assay. Three independent assays were performed. The results were expressed as the mean ± SD (*, *P* < 0.05; **, *P* < 0.01; ***, *P* < 0.001, #, *P* < 0.05; ##, *P* < 0.01; ###, *P* < 0.001, Student’s *t*‐test). (B) The estimation of IC50 values of cisplatin in the indicated gastric cancer cells treated with stattic. The results were expressed as the mean ± SD (*, *P* < 0.05; **, *P* < 0.01; ***, *P* < 0.001, Student’s *t*‐test). (C) Colony formation assay was used to determine growth inhibition of gastric cancer cells with the treatment of cisplatin or combination of cisplatin and stattic for 2 weeks. (D) qPCR analysis of multidrug‐resistance gene expression in ZIPK‐overexpressed and the respective control cells with the treatment of stattic. (E) qPCR analysis of multidrug‐resistance gene expression in ZIPK‐silenced and the respective control cells with the treatment of stattic. The results were expressed as the mean ± SD(*, *P* < 0.05; **, *P* < 0.01; ***, *P* < 0.001, Student’s *t*‐test)

To investigate the roles of ZIPK‐induced IL‐6/STAT3 pathway activation in chemotherapy resistance, STAT3 inhibitor stattic was used to deplete STAT3 activity. As shown in Fig 4, stattic strongly reduced STAT3 phosphorylation and the mRNA levels of IL‐6 in gastric cancer cells with high ZIPK expression. The gastric cancer cells were pretreated with stattic for 30 h followed by cisplatin. The relative sensitivity to cisplatin was detected by CCK‐8 assay. Inhibition of STAT3 pathway significantly potentiated cisplatin‐induced cell death in gastric cancer cells. The dramatical decreases in IC50 values were observed in ZIPK‐expressed cells treated with statitc (Fig. 5A,B). Colony formation assay indicated that stattic also strongly reduced the number and size of colonies in ZIPK‐expressed gastric cancer cells with cisplatin treatment (Fig. 5C). mRNA levels of multidrug‐resistance genes were attenuated after stattic treatment (Fig. 5D,E). These results indicated that IL‐6/STAT3 played an essential role in the modulation of ZIPK‐mediated CDDP resistance.

### Inhibition of STAT3 down‐regulated CDDP resistance gene expression in ZIPK‐expressed gastric cancer cells

**FIGURE 6:**
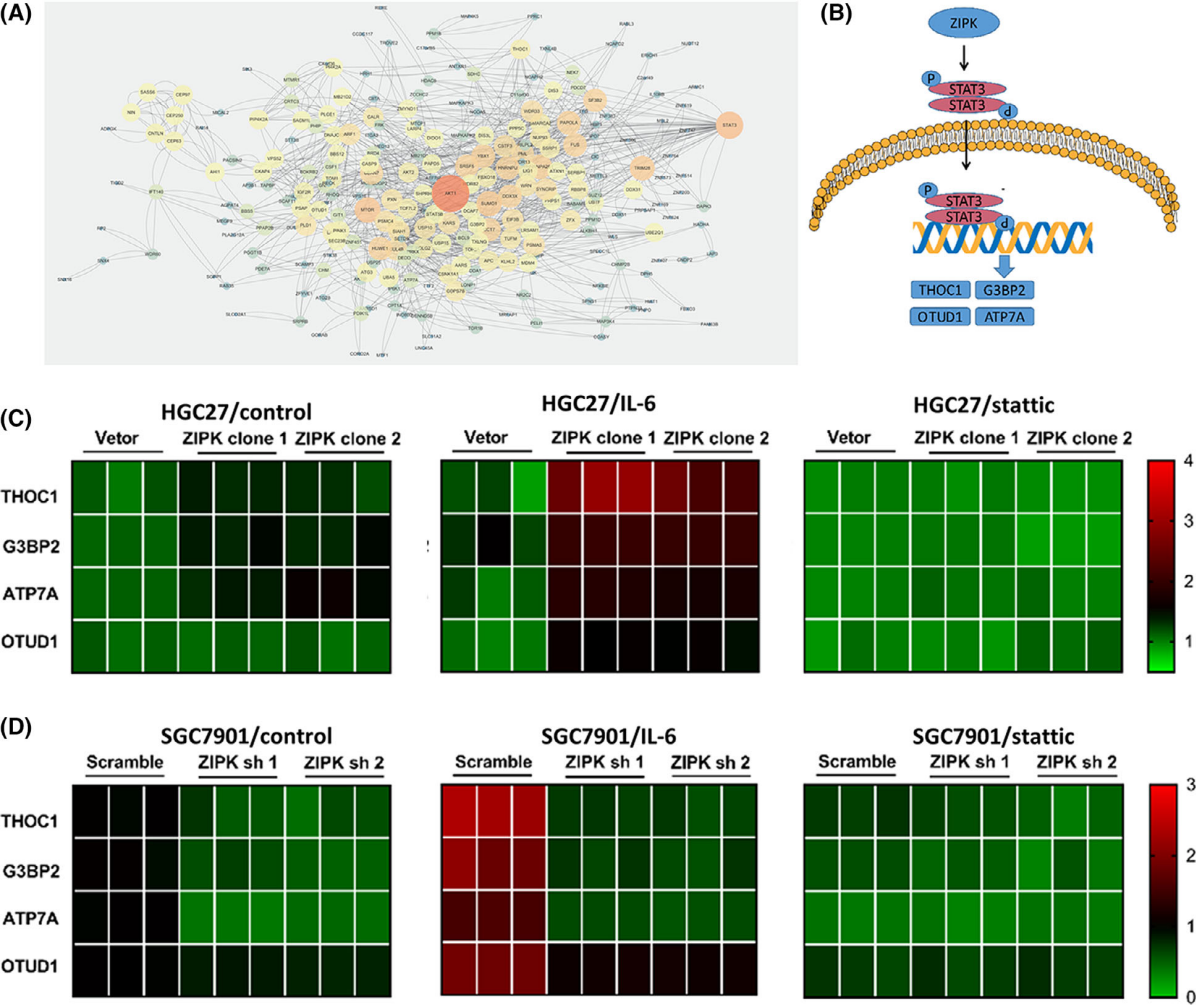
Inhibition of STAT3 down‐regulated CDDP resistance gene expression in ZIPK‐expressed gastric cancer cells. (A) The protein–protein interaction (PPI) network of STAT3‐related cisplatin‐resistant genes. The color and size of the bubbles indicated the degree of connectivity. (B) Schematic summary of the role of ZIPK in activating IL‐6/STAT3 signaling pathway and regulating the transcription of potential downstream genes. (C) Heat map of the mRNA expression levels of STAT3‐related cisplatin‐resistant genes in ZIPK‐overexpressed and control cells with the treatment of IL‐6 or stattic. (D) Heat map of the mRNA expression levels of STAT3‐related cisplatin‐resistant genes in ZIPK‐silenced and control cells with the treatment of IL‐6 or stattic

To determine the ZIPK‐related CDDP resistance gene expression, we first used public data from the TCGA database (TCGA‐STAD) and GEO database (GSE31811) to identify differentially expressed genes affected by STAT3 and genes related to S‐1, cisplatin, and docetaxel combination chemotherapy (DCS therapy) resistance, separately. A set of 7,263 genes associated with STAT3 and another set of 815 genes related to drug resistance were selected. By taking the intersection of the above two gene sets, we finally got 329 genes related to DCS therapy resistance and STAT3 expression and constructed a PPI network (Fig. 6A). To further narrow down the possible CDDP resistance genes, an extensive literature review was performed. These genes which were reported in cisplatin resistance were screened out. We found THOC1, G3BP2, ATP7A, OTUD1, PXN, and SREBP1 had the potential to be the downstream genes of STAT3 (Fig. 6B). To confirm the bioinformatics results, real‐time quantitative PCR was used to assess the expression of THOC1, G3BP2, ATP7A, OTUD1, PXN, and SREBP1 in ZIPK‐overexpressed cells, ZIPK‐silenced cells, and their control cells. qPCR assay showed that ZIPK promoted the mRNA expression levels of THOC1, G3BP2, ATP7A, and OTUD1, whereas knockdown of ZIPK dramatically weakened the mRNA expression of these genes. As expected, inhibition of STAT3 by stattic attenuated the expression of these CDDP resistance genes (Fig. 6C and D). Collectively, these findings suggested that ZIPK regulated CDDP resistance‐related gene expression through STAT3 pathway activation.

## Discussion

Chemotherapy resistance remains a significant obstacle in the treatment of gastric cancer. Our data indicated that ZIPK regulated CDDP resistance in GC cells through the activation of the IL6/ STAT3 signaling pathway. Application of STAT3 inhibitor could improve CDDP efficacy. Our finding indicated that CDDP in combination with STAT3 inhibitor might serve as a suitable treatment strategy for GC patients with ZIPK expression.

ZIPK is considered as a tumor suppressor because of its proapoptotic function [15]. However, recent studies show that ZIPK promotes cancer cell proliferation and migration. These findings suggest that the functional outcome of ZIPK may be context‐dependent. It may be attributed to the diversity of cells, tissues, and cancer types. Several lines of evidence indicate that ZIPK regulates a variety of signaling pathways which are commonly deregulated in cancer. ZIPK acts as a positive transcriptional regulator of Wnt signaling through the restoration of NLK‐mediated repression of Wnt signaling. ZIPK increases the expression of Wnt pathway downstream gene cyclin D1 and survivin and enhances colon cancer cell SW480 growth [27]. ZIPK is also a novel coactivator of the androgen receptor (AR) and has a crucial role in the regulation of degradation of the AR via ubiquitination and induction of efficient AR‐mediated transcription [11]. ZIPK promotes cell cycle progression, proliferation, cell growth, and invasion in nonsmall cell lung cancers. Moreover, ZIPK enhanced ERK/MAPK/c‐Myc pathway [18]. Our previous study demonstrated the pro‐oncogenic capabilities of ZIPK in gastric cancer cells cell lines. Further analysis indicated that ZIPK activated the AKT/IκB/NF‐κB pathway, which can promote EMT and metastasis. Our present study showed that ZIPK regulated CDDP resistance in SGC‐7901 and HGC27 GC cells through activation of the IL‐6/ STAT3 signaling pathway. Taken together, our and other studies imply that ZIPK regulates multiple signaling pathways to facilitate GC cell growth and progression.

STAT3, a member of signal transducers and activators of transcription (STAT) family of transcription factors, regulates various cellular function, including embryogenesis, inflammation, and immunity [28, 29]. Despite its crucial role in the physiological processes, STAT3 also promotes tumor development and progression. Aberrantly elevated STAT3 activity has been found in the majority of cancer, including gastric cancer. Constitutive STAT3 activation drives the key target genes transcription and subsequently accelerates cell proliferation, survival, angiogenesis, and metastasis [30, 31, 32]. For instance, the expression of VEGF and MMPs induced by activated STAT3 facilitates angiogenesis and invasiveness [23]. A large body of evidence illustrates that STAT3 has a contributing role in cellular multidrug resistance. Constitutive activation of STAT3 and overexpression of its target gene Bcl‐2 and c‐Myc are detected in cisplatin‐resistant gastric cancer cells. Inhibition of the STAT3 pathway remarkably restores the sensitivity of gastric cancer cells to chemotherapeutic agents and increases apoptosis in drug‐resistant cells [33]. STAT3 is activated through Tyr‐705 phosphorylation by tyrosine kinases such as JAKs and Src family members and growth factor receptors [34, 35]. Phosphorylation of STAT3 at Ser727 induced by several kinases, including JNK, MAPKs, and protein kinase C, also enhances STAT3 activity [36]. Phosphorylation of STAT3 in DNA binding complex facilitates the complex to the nucleus, binds DNA, and thereby activates transcription [37]. Binding of IL‐6 to IL‐6R on the cell surface initiates JAK‐mediated STAT3 activation. STAT3 also interacts with IL‐6 promoters and increases IL‐6 transcription. NF‐kB and IL‐32R are required for the interaction [23, 38, 39]. Several lines of evidence demonstrate that the autocrine/paracrine IL‐6/JAK/STAT3 feed‐forward loop participates in tumor progression and shapes of the tumor microenvironment [26]. The interaction of STAT3 and ZIPK has been identified in mammalian cells. ZIPK phosphorylates STAT3 on Ser727 and activates IL‐6‐induced STAT3‐dependent transcription [40]. In our present study, we demonstrated that ZIPK activates IL‐6/STAT3 signaling pathway via Ser727 phosphorylation in gastric cancer cells. Effective blockade of the STAT3 activity by STAT3 inhibitor sensitized gastric cancer cells to cisplatin and reduced the cisplatin‐resistant‐related gene G3BP2, THOC1, ATP7A, and OTUD1 expression [41, 42, 43, 44, 45]. Our findings suggest that ZIPK plays a pivotal role in chemotherapy resistance, and inhibition of STAT3 might be a potential strategy to overcome GC treatment failure.

## Conflict of interest

All authors have completed the ICMJE uniform disclosure form. The authors have no conflict of interest to declare.

## Author contributions

(I) Conception and design: JB; (II) Administrative support: QS,JB and XH; (III) Provision of study materials or patients: GL and ZD; (IV) Collection and assembly of data: HF and QO; (V) Data analysis and interpretation: HF,QO, and QS; (VI) Manuscript writing: HF and JB; (VII) Final approval of manuscript: All authors.

## Supporting information

**Fig**. **S1**. The expression of ZIPK in stable cell lines.
**Fig**. **S2**. The sensitivity to cisplatin in HGC27 and SGC7901 cell lines.
**TableS1**. Sequences of primers used in this study.
**TableS2**. Reagent source used in this study.

## Data Availability

The dataset to identify differentially expressed genes affected by STAT3 is available in TCGA (https://portal.gdc.cancer.gov/). The other dataset identifying genes related to S‐1, cisplatin, and docetaxel combination chemotherapy (DCS therapy) resistance is available in the GEO database at https://doi.org/10.1159/000464329, reference number22.
