# Supplementary material for: ZIPK activates the IL‐6/STAT3 signaling pathway and promotes cisplatin resistance in gastric cancer cells
Source: FEBS Open Bio. 2021 Aug 22;11(9):2655–67. doi: 10.1002/2211-5463.13270 (PMC8409285; doi:10.1002/2211-5463.13270)
Supplement: Supplementary file 1 — Fig. S1. The expression of ZIPK in stable cell lines. Fig. S2. The sensitivity to cisplatin in HGC27 and SGC7901 cell lines. TableS1. Sequences of primers used in this study. TableS2. Reagent source used in this study. [file FEB4-11-2655-s001.docx]

SUPPLEMENTARY FIGURE AND TABLES


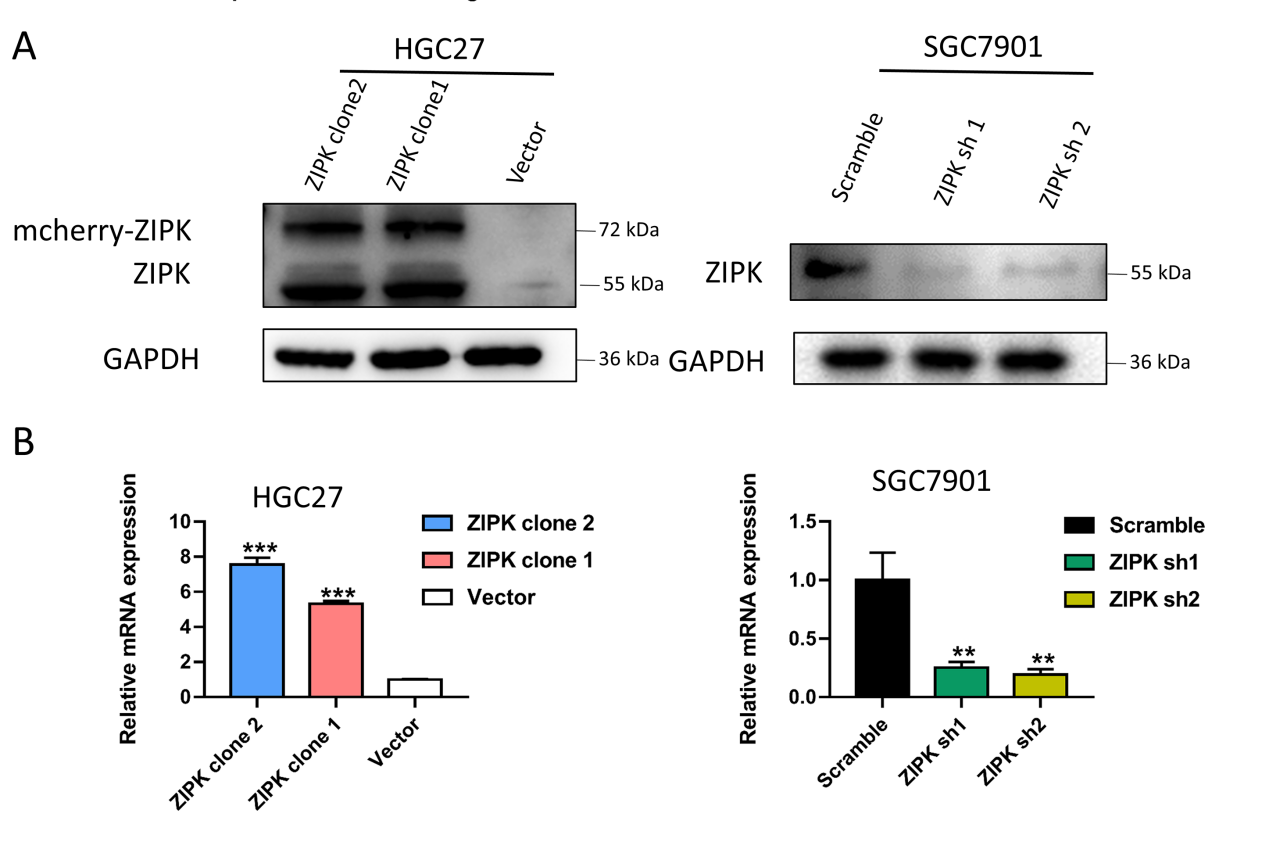


Supplementary Figure 1: (A) Expression of ZIPK was analyzed by Western blotting in Stable ZIPK-overexpressed cell clones from HGC27 cell line. The molecular weight of endogenous ZIPK was 52 kDa, and ectopic expression of mcherry -tagged ZIPK was 80 kDa. The protein levels of ZIPK was tested by Western blotting in Stable ZIPK- silenced cell clones from SGC7901 cell line. GAPDH was used as a loading control. (B) ZIPK mRNA level was detected in ZIPK-overexpressed, ZIPK-knockdown, and their respective control cells by qRT-PCR (*Significant difference between the control group and the rest groups, independent Student’s t-test).


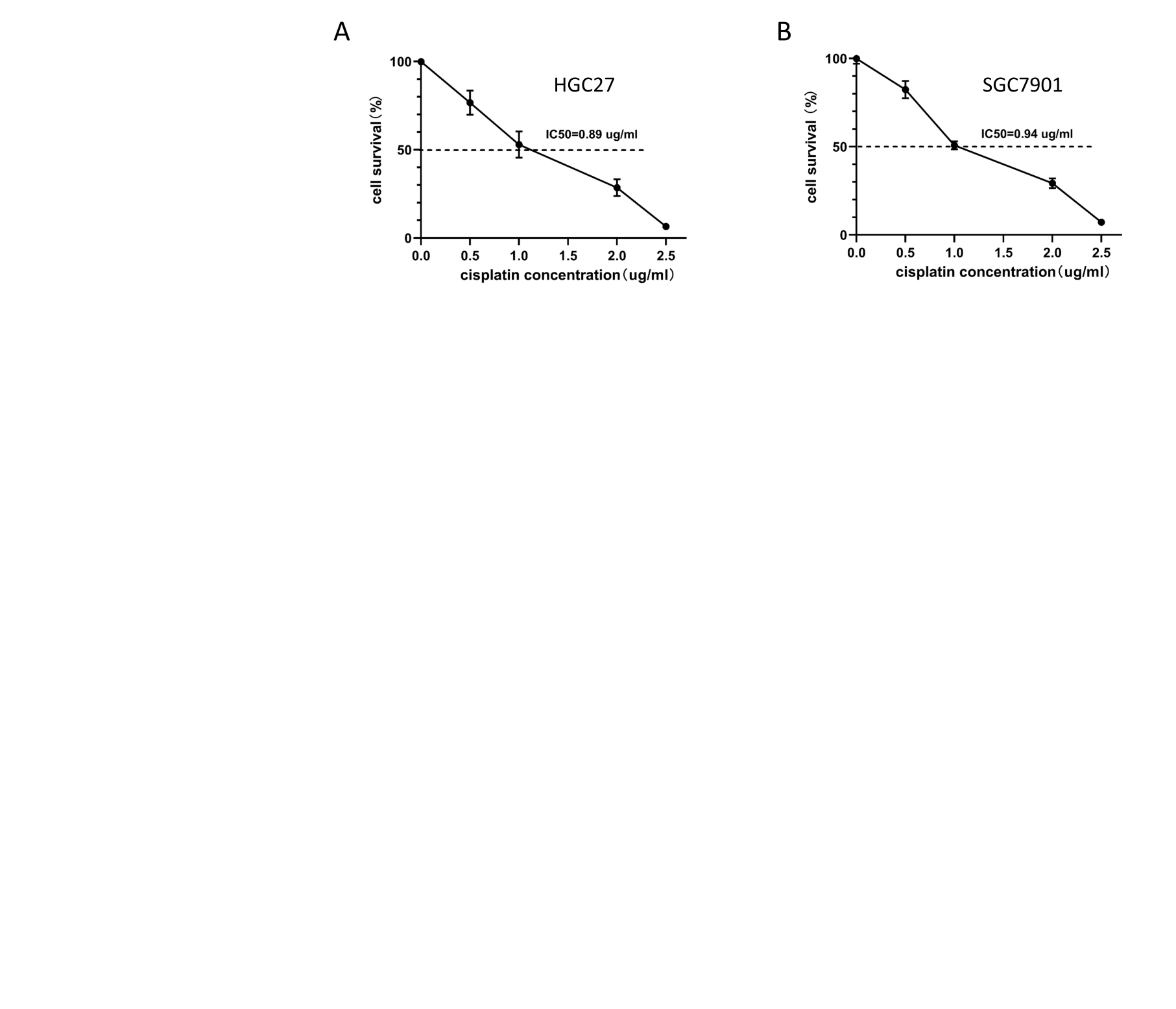


Supplementary Figure 2: The sensitivity to cisplatin in HGC27（A） and SGC7901 （B） cell lines were detected by CCK-8 assay.

Supplementary Table 1 : Sequences of primers used in this study

| **Gene** | **Forward primer (5ʹ-3ʹ)** | **Reversed primer (5ʹ-3ʹ)** |
| --- | --- | --- |
| **IL6** | CCAGGAGCCCAGCTATGAAC | CCCAGGGAGAAGGCAACTG |
| **ATP-7A** | TGTGTGCAGTCTATTGAGGGT | TGACAAGGTAGCATCAAATCCC |
| **ABCB1** | TTGATGGCAAAGAAATAAAGC | CTTACATTAGGCAGTGACTCG |
| **ABCG2** | GCACCTCAACCTGCCCATT | TCAGGGTGCCCATCACAAC |
| **THOC1** | TCTTCTGTGGACGGATTCAGC | CTCGTCTCCCATTTCGCCTTC |
| **G3BP2** | GTAGGGCGGGAGTTTGTGAG | CTGGGGCTTTCCACTAGCATC |
| **OTUD1** | GACGAGAAGCTGGCCCTATAC | TGGAATGATGTGGAATCGGTACT |
| **MRP1** | AAGGGATCGCCGTGTTTGG | CGAAGGCTCGAATGACGCTGAC |
| **MVP** | TTTGATGTCACAGGGCAAGTTCGGC | CACCAAATCCAGAACCTCCTCAAAC |
| **ZIPK** | GAGGACCATTATGAGATGGGGG | CTCCCGCAGGATGTTCACC |
| **GAPDH** | GCACCGTCAAGGCTGAGAAC | TGGTGAAGACGCCAGTGGA |

Supplementary Table 2:Reagent source used in this study

| **REAGENT or RESOURCE** | **SOURSE** | **IDENTIFIER** |
| --- | --- | --- |
| **Antibody** |  |  |
| **STAT3** | Cell Signaling Technology | 9139, RRID:AB_331757 |
| **STAT3-Ser727** | Cell Signaling Technology | 9134, RRID:AB_331589 |
| **ZIPK** | ProSci | APO-2067, RRID:AB_10692263 |
| **GAPDH** | Bioss | bs-2188R, RRID:AB_10856675 |
| **Chemicals and Recombinant Proteins** |  |  |
| **STATTIC** | MCE | [HY-13818](https://www.medchemexpress.cn/Stattic.html) |
| **IL-6** | Novoprotein | C691 |
